# Environmentally-driven gene content convergence and the *Bacillus* phylogeny

**DOI:** 10.1186/s12862-018-1261-7

**Published:** 2018-10-03

**Authors:** Ismael L. Hernández-González, Gabriel Moreno-Hagelsieb, Gabriela Olmedo-Álvarez

**Affiliations:** 1 Department of Genetic Engineering, CINVESTAV-Irapuato, Km. 9.6 Libramiento Norte, Carr. Irapuato-Leon, Irapuato, 36824 Guanajuato Mexico; 2 Department of Biology, Wilfrid Laurier University, 75 University Ave. W., Waterloo, N2L 3C5 Ontario Canada

**Keywords:** Bacillus evolution, Phylogenomics, Comparative genomics, Evolutionary genomics, Functional content, Homoplasy

## Abstract

**Background:**

Members of the *Bacillus* genus have been isolated from a variety of environments. However, the relationship between potential metabolism and the niche from which bacteria of this genus have been isolated has not been extensively studied. The existence of a monophyletic aquatic *Bacillus* group, composed of members isolated from both marine and fresh water has been proposed. Here, we present a phylogenetic/phylogenomic analysis to investigate the potential relationship between the environment from which group members have been isolated and their evolutionary origin. We also carried out hierarchical clustering based on functional content to test for potential environmental effects on the genetic content of these bacteria.

**Results:**

The phylogenetic reconstruction showed that *Bacillus* strains classified as aquatic have evolutionary origins in different lineages. Although we observed the presence of a clade consisting exclusively of aquatic *Bacillus*, it is not comprised of the same strains previously reported. In contrast to phylogeny, clustering based on the functional categories of the encoded proteomes resulted in groups more compatible with the environments from which the organisms were isolated. This evidence suggests a detectable environmental influence on bacterial genetic content, despite their different evolutionary origins.

**Conclusion:**

Our results suggest that aquatic *Bacillus* species have polyphyletic origins, but exhibit convergence at the gene content level.

**Electronic supplementary material:**

The online version of this article (10.1186/s12862-018-1261-7) contains supplementary material, which is available to authorized users.

## Background

The heterogeneous *Bacillus* genus consists of rod-shaped gram-positive bacteria that can be either aerobic or facultative anaerobic. These bacteria are capable of forming endospores, which have a high resistance to heat and desiccation. Members of the *Bacillus* genus are ubiquitous in nature and have been isolated from a large variety of terrestrial and aquatic environments. However, it has been intriguing whether their presence in such environments is due to spore dispersion by air and water or due to their metabolic capabilities [1].

The importance of *Bacillus* species in medicine, in the environment, and in industrial applications [2] is reflected in the large number of genome sequences deposited in the GenBank database. Over 2000 complete and *draft* genome sequences belonging to this genus had been deposited in the GenBank database by November 2017. A majority of these *Bacillus* sequences are from strains either of medical relevance, or isolated from terrestrial environments. However, the number of publicly available genome sequences of *Bacillus* isolated from aquatic environments (marine and fresh water) has recently increased. Phenotypic and molecular analysis of a phylogenetically diverse marine sediment sample uncovered the existence of a strict marine group [3]. In addition, a phylogenomic reconstruction from 20 complete genomes suggested the existence of a monophyletic group consisting exclusively of *Bacillus* from aquatic environments [4]. With additional genomes available today, we wondered if the aquatic *Bacillus* group would still hold and whether there is a relationship between gene content and the different environments from which the different strains have been isolated.

The remarkable variability of the gene repertoire in bacteria, represented in the so-called accessory (or flexible) genome, provides potential adaptability to access different ecological niches. Diversification is facilitated by the movement of genes through lateral gene transfer which is aided by phages, plasmids, pathogenicity islands, and insertion elements. Genes that are not vertically inherited are a source of noise when constructing phylogenies [5, 6]. The process of creating an accessory genome has been extensively explored in pathogens. In these, virulence and pathogenicity genes, as well as antibiotic resistance traits, are commonly found, and are often the result of lateral gene transfer [5, 7]. The phenotypic or genotypic characteristics that are shared by a set of organisms, but not inherited from a common ancestor are referred to as homoplasies [8] and are suggestive of convergent evolution.

We evaluated the relationship between the evolutionary history of *Bacillus* species and their environments of isolation. We investigated whether the aquatic *Bacillus* clade described by Alcaraz et al. [4] would hold with a larger genome sequence sample, and if the aquatic *Bacillus* species indeed have a monophyletic origin. We gathered information on the environments from which these *Bacillus* were isolated from several sources. To study if the environment has selected for genes with particular functions, we also compared the gene content of these genomes and tested for an association between gene functional annotations and the environment from which these *Bacillus* strains have been isolated.

## Methods

### Screening and selection of genome sequences

Genomic sequences were obtained from NCBI’s RefSeq genome database [9] (the genomes sequenced by our group are also available in RefSeq). To organize the complete *Bacillus* genomes into single-“species” clusters, we used a web-based tool [10] using a Genome Similarity Score *GSSa*≥0.95 [10] (http://microbiome.wlu.ca/research/redundancy/redundancy.cgi). We chose two strains from clusters containing more than one member and the one strain from every single-strain cluster. Because we were particularly interested in aquatic *Bacillus*, we manually selected some “draft” genomes that included aquatic species not reported in the complete genome data set.

### Evolutionary analysis using 16S rRNA gene sequences

To choose a representative 16S rRNA sequence, out of the multiple copies present in each *Bacillus* genome, we clustered the 16S rRNA genes of each genome at 97% identity (the threshold usually used to define operational taxonomic units in bacteria) using the CD-HIT-EST program [11]. We selected the 16S rRNA sequence that the CD-HIT program returned as the representative of the cluster containing the largest number of sequences. A structure-based alignment of the selected 16S rRNA gene sequences was built using Infernal [12]. The alignment was probabilistically masked using Zorro [13], and the mask used to trim the alignment. The final alignment consisted of 1541 positions. To build the phylogenies based on these genes, the substitution model was determined using jModelTest2 [14] (version 2.1.10). The phylogenetic reconstruction was performed by Maximum Likelihood (ML) with PhyML [15] (version 3.3.2017080) and the substitution model GTR+I+G+F.

### Phylogenomic reconstruction based on marker sequences

For each genome in our data set, phylogenomic markers were searched at the protein sequence level using the AMPHORA2 package [16]. Eleven markers found in each genome were aligned and trimmed based on the models and masks provided by AMPHORA2 using the script *MarkerAlignTrim.pl* [16]. The trimmed and aligned marker genes were concatenated to build a super matrix of 2088 residues. The ProtTest3 program [17] (version 3.4.2) and a Maximum Likelihood (ML) phylogenetic reconstruction was built using PhyML [15] with the substitution model LG+I+G.

### Core genome phylogenies and *GSS*

The “core genome” is commonly defined as the set of genes shared by all the strains in a single bacterial species [18, 19]. This concept has been extended to embrace other taxonomic levels [20]. To select the protein coding sequences that comprise the core genome in our data set, we selected orthologs as BLAST reciprocal best hits (RBHs), as previously described [21, 22]. We filtered these RBHs to include alignments covering at least 70% of the aligned proteins. The core genome consisted of RBHs present in all the genomes in the data set. These sequences were aligned using Muscle [23] (version 3.8.1551). To reduce uncertainty in the alignment, each alignment was probabilistically masked using Zorro [13], and the mask used to trim each alignment. Trimmed alignments were concatenated to get a super-matrix with 62,590 amino acids. ProtTest3 computed the best-fit substitution model [17]. The LG+I+G+F substitution model was used to perform the phylogenomic reconstruction by ML using PhyML [15].

Since genes other than those shared by all genomes can also be inherited vertically, we also estimated a phylogenetic tree based on a “Core 70” protein dataset, meaning that we used orthologous proteins, again as RBHs, encoded by genes present in at least 70% of the genomes under analysis. The Core 70 dataset contained 437 ortholog protein sets. These were treated in the same way as the core dataset. The Core 70 concatenated amino acids data matrices were analyzed by ML using the RAxML program (arguments: -m PROTGAMMALGF -# 100) [24] implemented in the CIPRES *Science Gateway* platform (Cyberinfrastructure for Phylogenetic Research) [25].

Additionally, the genomic similarity score (*GSSa*) was calculated between all of the organisms in our data set as previously described [10, 26]. A neighbor-joining tree was computed using the *GSS* distance matrix [4] and the neighbor program from the Phylip software suite [27] (version 3.3.20170530). The reliability of the branches in the *GSS*-tree topology was estimated, based on maximum likelihood, using the software WeightLESS [28].

### Tree congruence

The congruence between the topologies of the trees and clusters was computed as Robinson-Foulds Symmetric Differences [29] using the Treedist program included in the Phylip package [27].

### Environmental classification

The environment for each organism in our data set was determined using several sources, including NCBI’s RefSeq genome database [9], the HAMAP database [30] (the environment of isolation is no longer annotated in this database), PATRIC [31], and, failing the above, the scientific literature. To classify the bacterial strains into their natural environments, we used the same criteria as Parter et al. [32]. Thus, the *Bacillus* species were classified in the following manner: those isolated from fresh or marine water were called *Aquatic*; those isolated from soil were called *Terrestrial*; those found to be free-living or associated to a hosts were called *Facultative*; and those isolated from thermal vents or air, were called *Specialized*. We added the *Unknown* category for strains we could not classify into any of the previous categories. The procedure classified the bacteria into 26 Terrestrial, 23 Facultative, 18 Aquatic, seven Unknown, and three Specialized strains (Additional file 1: Table S1).

### Functional content

To investigate whether *Bacillus* species living in similar environments share genes with common functions, we found and compared the functional content of each genome under study. The functional content was defined using COGs (Cluster of Orthologous Groups) [33] and Figfams [34].

The COG classifications for the proteins in our genome data set were assigned using the RPSBLAST program [35], and the COG position-specific score matrices (PSSMs) [35]. The COG assignments were filtered to eliminate those with alignments covering less than 70% of the PSSMs. We allowed a maximum overlap between aligned COGs within a protein to be ≤ 10%.

To obtain functional content using Figfam categories, we used RAST (Rapid Annotations using Subsystems Technology) annotations [36] obtained from the PATRIC database [31]. Genomes not present in PATRIC were annotated using the myRAST Toolkit interface.

To compare genomic functional contents, Jaccard indices were computed: *J*(*A*,*B*)=(*A*∩*B*)/(*A*∪*B*) (where A and B are the COG/Figfam sets present in genomes A and B, respectively), and Jaccard distances were determined using *Jd*(*A*,*B*)=1−*J*(*A*,*B*). The matrices containing Jaccard distances (*Jd*) for COG and Figfams were each used to carry out hierarchical clustering testing several agglomerative methods.

The quality of the hierarchical structures was measured using the agglomerative coefficient. This coefficient is defined as one minus the average ratio of dissimilarity of one unit to the cluster with which it first merges to the dissimilarity of its merging in the final step. If the coefficient is close to zero, the algorithm did not find a natural cluster structure (the data consist of a single cluster). However, if the coefficient is close to one, then clear clusters formed.

The clusters obtained were further evaluated based on their Silhouette values [37]. These reflect the distances of each unit of a cluster to the other units of the same cluster, compared to the distances against members of other clusters. The values range from -1 to 1, wherein 1 means that the unit clearly belongs in the cluster where it landed.

### Association between natural environment and genome clusters

To test for a potential association between the natural environments and the clusters obtained in the COG and Figfam dendrograms, we cut the COG and Figfam dendrograms into smaller clusters and calculated *p*-values for each resulting cluster based on the hypergeometric distribution. The resulting *p*-values were corrected by employing a False Discovery Rate (FDR) with the p-adjust function as implemented in R [38]. The cuts were performed starting with *k*=3 and continued cutting into smaller groups until the significant *p*-values were lost. The COG dendrogram was thus cut to a maximum of *k*=22, and the Figfam dendrogram to a maximum of *k*=16 clusters.

The search of group-specific functions, or potentially adaptive COGs/Figfams, was performed using the ShotgunFunctionalizeR package [39] from R. This package allows the comparison of groups with multiple samples and computes corrected *p*-values. We compared domain frequencies between all pairs of groups and sought for domain families with significant *p*-values. We used a corrected *p*-value ≤0.05 and confirmed that the COGs/Figfams were present in at least 80% of each group. Then, we checked whether the family was underrepresented or overrepresented with respect to other groups.

### Statistical analyses

All the statistical analyses were performed using the free software environment for statistical computing and graphics R [38].

## Results and discussion

**Fig. 1:**
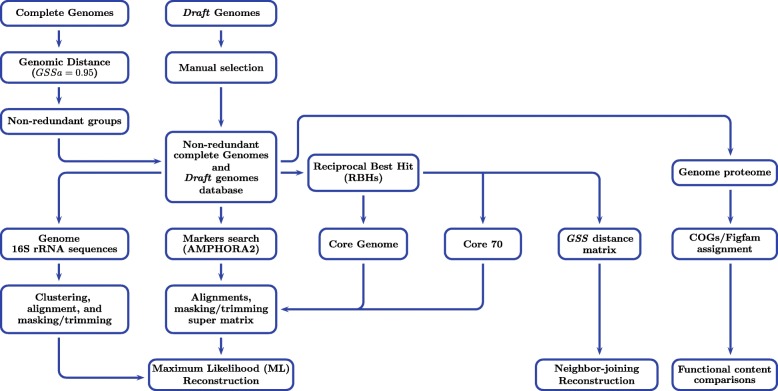
Overview of the methodology used in this work

To investigate the potential relationship between the evolutionary origins of the *Bacillus* genus members and their environments, we carried out phylogenetic reconstructions and compared them with information about their natural environments (see Fig. 1 for an overview of the study).

### Genome selection

The representative genome selection process resulted in 50 complete genomes selected from 30 species-like clusters (see “Methods” section), and 29 draft genomes. The latter included five *Bacillus* genomes recently sequenced by our group. Additionally, four species within the family *Bacillaceae*, but outside of the genus *Bacillus*, were included: one species of the genus *Oceanobacillus*, one of *Geobacillus*, and two of the genus *Listeria*. While all four species were originally included as an outgroup set, our results (Fig. 3), along with results from another study [40], have suggested that both *Oceanobacillus*, and *Geobacillus*, belong to the *Bacillus* genus. Thus, our data set contained 83 species: 79 *Bacillus* genomes, two genomes that despite their current classification might belong in the *Bacillus* genus, and two *Listeria* genomes serving as an outgroup (Additional file 2: Table S2).

### Evolutionary origins of the aquatic *Bacillus*

To study the phylogenetic relationships between the gathered *Bacillus* genomes, we carried out several phylogenetic reconstructions using Maximum Likelihood (ML). We produced a phylogenetic tree using 16S rRNA gene sequences, a phylogenomic tree using the concatenated alignments of 11 AMPHORA2 phylogenomic markers conserved in every *Bacillus* species in our data set, a phylogenomic tree using the concatenated alignment of the proteins encoded by the core genes in our genomic dataset, and a “Core 70” phylogenomic tree based on proteins encoded by genes present in at least 70% of the genomes.

The Maximum Likelihood phylogeny using the 16S rRNA sequence resulted in a poorly supported tree as reflected in the low number of clades with bootstrap values ≥80*%* (Table 1). The tree revealed two major groups inside the genus, those of *B. cereus* and *B. subtilis*, but showed low support in the internal nodes (Additional file 3: Figure S1).

**Table 1:** Number of nodes with bootstrap values ≥80

	16S rRNA	AMPHORA markers	Core genome	Core 70	*GSS* distance*
Num. Nodes ≥80	22	44	69	74	46

^*^The GSS node quality is based on maximum likelihood (*p*≤0.05), not bootstrap analysis

The phylogenetic reconstruction using the 11 AMPHORA2 markers present in the genomes analyzed, resulted in a better-resolved tree than that obtained with the 16S rRNA sequences. The 11 markers tree had a higher number of clades supported by bootstrap values ≥80*%* (Table 1). The *B. cereus* and *B. subtilis* groups observed in the 16S rRNA tree were also found in this phylogeny. Five groups were better supported in the 11 marker tree than in the 16S rRNA tree, including: *B. megaterium*, *B. clausii*, *B. methanolicus*, *B. coagulans* and *B. isronensis* (Additional file 4: Figure S2).

The phylogenetic tree based on the core genome [41] contained 196 orthologous groups. The number of genes in the core genome was fewer than the 814 genes found by Alcaraz et al. [4], as expected, since the number of shared genes tends to decrease with the number of strains under analysis [18]. The core tree contained a higher number of nodes supported by bootstrap values ≥80*%* (Table 1).

The Core 70 tree, produced from 437 orthologous groups, contained a higher number of nodes supported by bootstrap values ≥80*%* than the core genome tree (Table 1) and a very similar topology (Table 2).

**Table 2:** Symmetric differences between trees

	16S rRNA	AMPHORA markers	Core genome	Core 70	*GSS* distance
16S rRNA	0	-	-	-	-
AMPHORA markers	112	0	-	-	-
Core genome	102	64	0	-	-
Core 70	100	70	10	0	-
*GSS* distance	102	72	32	24	0

**Fig. 2:**
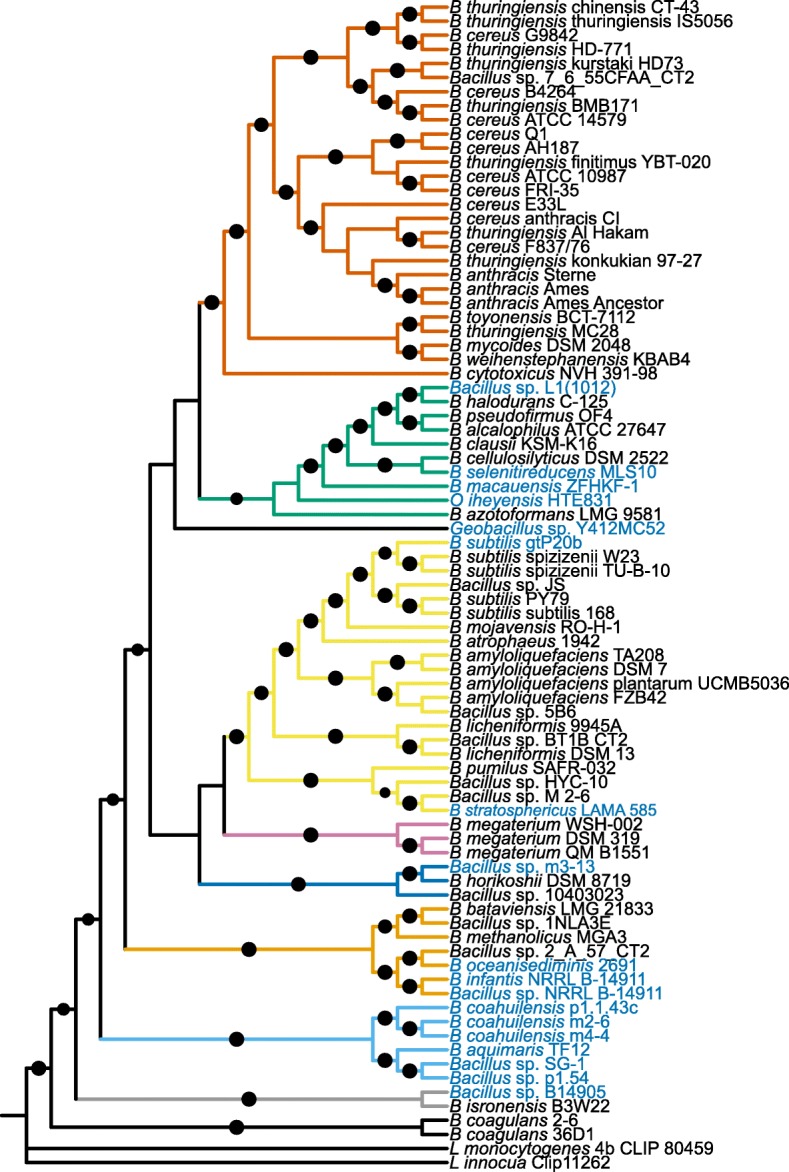
Phylogenetic reconstruction based on 196 protein orthologous groups comprising the Core Genome. Note the well supported nodes. Branch colors indicate the nine main groups discussed in the text. The names of the species corresponding to *Bacillus* isolated from aquatic environments are shown in light blue. Bootstrap values ≥80 are indicated by dots. The figure was constructed using the web-based tool iTOL [68]

Visual inspection of the core-genome phylogenomic reconstruction revealed nine well-supported main clades (Fig. 2). The largest clade featured species in the *B. cereussensu lato* group [42–44] including *B. cereus*, *B. thuringiensis*, *B. anthracis*, *B. weihenstephanensis*, *B. mycoides*, *B. toyonensis*, and *B. cytotoxicus*. The short distances observed between the organisms in this clade confirmed that they were closely related, as has been previously established in studies using multilocus enzyme electrophoresis (MEE) [42], multilocus sequence typing (MLST) [43, 45] and genomic comparisons [46, 47].

**Fig. 3:**
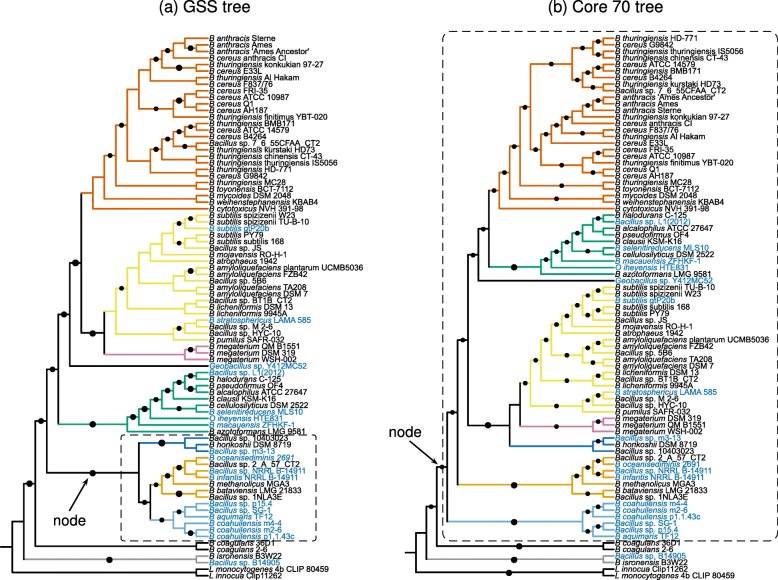
Comparison of Genomic Similarity Score and Core 70 trees. **a** Neighbor-joining tree based on *GSS* (Genomic Similarity Score) distance [10]. **b** Core 70 tree based on the alignment of 437 genes present in at least 70*%* of the species. In both trees, strain names in blue correspond to *Bacillus* species isolated from aquatic environments. Branch colors indicate clearly delineated groups. The dots indicate support based on *p*≤0.05 (GSS tree) and bootstrap values ≥80 (Core 70 tree). Indicated nodes and dashed rectangles show that aquatic *Bacillus* cluster better in the GSS tree. The figure was drawn using the web-based tool iTOL [68]

The second largest clade included the species *B. subtilis*, *B. atrophaeus*, *B. mojaviensis*, *B. amyloliquefaciens*, *B. licheniformis*, and *B. pumilus*, as well as some *Bacillus* not yet classified at the species level. This clade included two marine isolates, *B. stratosphericus*, and *B. subtilis* gtP20b.

A third clade included mainly alkaliphylic species, such as *Bacillus*: *B. alcalophilus*, *B. pseudofirmus*, *B. halodurans*, *B. clausii*, *B. selenitireducens*, *B. cellulosilyticus*, *B. macauensis*, *B. azotoformans*, and the marine *Bacillus* sp. L1(2012). This clade showed longer internal nodes than other clades, suggesting that this clade was more diverse. *Oceanobacillus iheyensis* and *Geobacillus* sp. Y412MC52 were located inside and basal to this clade, respectively. However, their position was not well supported as their bootstrap values were <80*%* (Fig. 2).

The strains of *B. megaterium* formed a monophyletic group, located closest to the *B. subtilis* clade. Next to this, *B. horikoshii*, *Bacillus* sp. 10403023 and the aquatic *Bacillus* sp. m3-13 formed another clade. Meanwhile *B. methanolicus*, *B. bataviensis*, *Bacillus* sp. 1NLA3E and *Bacillus* sp. 2 A 57 CT2 clustered with the marine strains *B. oceanisediminidis*, *B. infantis* and *Bacillus* sp. NRRL B-14911. Basal to the latter clade, we observed a clade consisting exclusively of aquatic *Bacillus* isolated from marine and fresh water environments. This clade contains *B. aquimaris* TF12, *Bacillus* sp. SG1 and *Bacillus* sp. p15.4, as well as all the strains of *B. coahuilensis*. The last two clades are formed by *B. isronensis* and *Bacillus* sp. B14905, a marine strain. A final clade included *B. coagulans* strains, the *Bacillus* species with the smallest genome reported to date.

It is important to note that the previously found clade of aquatic *Bacillus* [4], was not obtained in our analysis, since the strains (*B. coahuilensis* m4-4, *Bacillus* sp. NRRL B-14911 and *Bacillus* sp. m3-13) were dispersed among three different clades (Fig. 2). The disruption of this clade might be due to the larger number of available strains isolated from diverse environments available for this study. The distribution in our tree thus suggested that the aquatic *Bacillus* were polyphyletic. Multiple clades held at least one aquatic *Bacillus*. However, we still observed a clade consisting exclusively of species isolated from aquatic environments (Fig. 2). These clustered aquatic strains, isolated from dissimilar geographic sites, support the hypothesis of a single evolutionary origin for at least some of the aquatic *Bacillus* species.

In general, all trees maintained similar major clades and differed in their number of well supported clades (Table 1). Despite differences in bootstrap support among the different trees, all trees showed similar distributions with respect to the polyphyletic origin of the aquatic *Bacillus* species. Furthermore, the clade formed exclusively of aquatic *Bacillus* species was present in all of our phylogenetic reconstructions (Fig. 2, Additional file 3: Figures S1 and Additional file 4: Figures S2).

Our results were mostly consistent with those obtained by Alcaraz et al. [4], as well as with results using the concatenated alignments of 157 single-copy genes focusing on the *Bacillaceae* family [48]. Our results were also similar to another work based on 20 housekeeping and ribosomal proteins of 34 species of *Bacillus* [49]. All of these results revealed the *B. cereus*, *B. subtilis* and *B. clausii*/*B. halodurans* as major clades.

### Differences between genomic similarity groups and phylogenetic clades suggests gene-content effects

In addition to our phylogenetic reconstructions, we computed the Genomic Similarity Score (*GSS*) distance matrix, as previously described [4], and built a tree using the neighbor-joining method (Fig. 3a). The *GSS* score was calculated by computing Reciprocal Best Hits between each pair of genomes, thus including information of the overall core genome and the shared accessory genome.

In contrast with the phylogenetic trees, the *GSS* tree showed a clade containing 10 of the 18 aquatic *Bacillus* genomes, mixed with only six non-aquatic genomes (Fig. 3a). The aquatic genomes are so spread in the core genome phylogeny that these ten genomes cannot be brought into a single clade without considering most of the tree (Fig. 2). The same is true for the Core 70 tree (Fig. 3a).

Since the *GSS* is based on the BLAST scores of every RBH shared by each pair of genomes, the clustering of aquatic genomes suggests higher shared gene content between aquatic organisms than would be suggested from their polyphyletic origins. This shared gene content might be explained by “convergent” lateral gene transfer. In other words, the shared gene content might have a homoplasic component perhaps influenced by the environment.

Lateral gene transfer can potentially introduce new functions into an organism [50, 51], and thus enable adaptation to new environments. Accordingly, metabolic convergence has been observed in endosymbionts, where bacteria from distinct phylogenetically origins have shown convergence toward similar functional profiles. For instance, the endosymbiont *Xiphinematobacter* from dagger nematode *Xiphinema americanum*, a migratory ectoparasite of numerous crops, showed evolutionary convergence with endosymbionts found in sap-feeding insects, possibly due to the similarity of their feeding mode [52]. Also, a high degree of metabolic convergence has been observed among very distantly related endosymbiotic bacteria of blood-feeders [53]. The adaptive convergence of horizontally transferred genes has also been observed in two human-restricted pathogens [54].

To test whether gene content was responsible for the better clustering of aquatic genomes found in the *GSS* tree, we decided to test clusters based on gene content as represented by functionally-annotated protein domains. The functional content, defined as the set of functions assigned through either Clusters of Orthologous Groups (COG) [33] or Figfam [34] categories, was determined for each genome. To compare functional contents we used Jaccard distances as a measure of functional similarity and evaluated different hierarchical clustering methods to choose the one producing the best dendrogram. The quality of clustering was measured using agglomerative coefficients (see “Methods” section). Essentially, the closer the agglomerative coefficient is to one, the better the hierarchy’s quality.

**Fig. 4:**
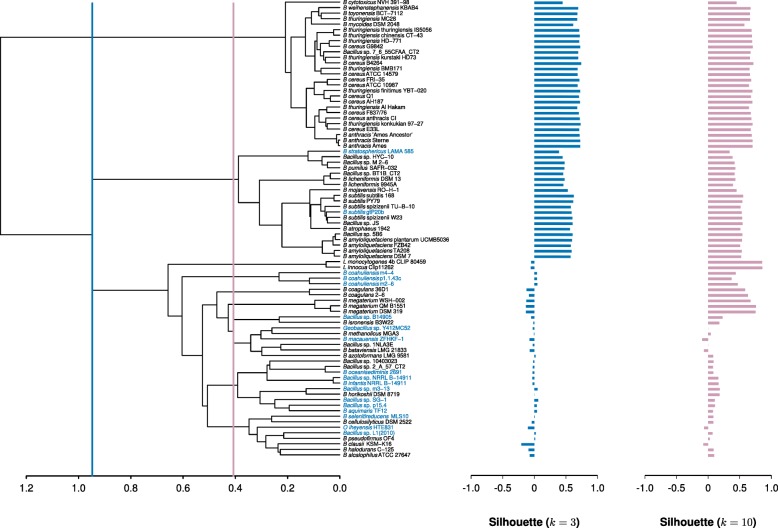
Hierarchical clustering using COGs-based Jaccard’s distance (Jd). The dendrogram was obtained using the COGs-based Jaccard’s distance and the Ward clustering method. The silhouette values are shown as blue and pink bars for *k*=3 and *k*=10, respectively. The blue and pink lines indicate the heights where the dendrogram was cut (*k*=3 and *k*=10, respectively). The names of the strains shown in light blue correspond to *Bacillus* isolated from aquatic environments

**Fig. 5:**
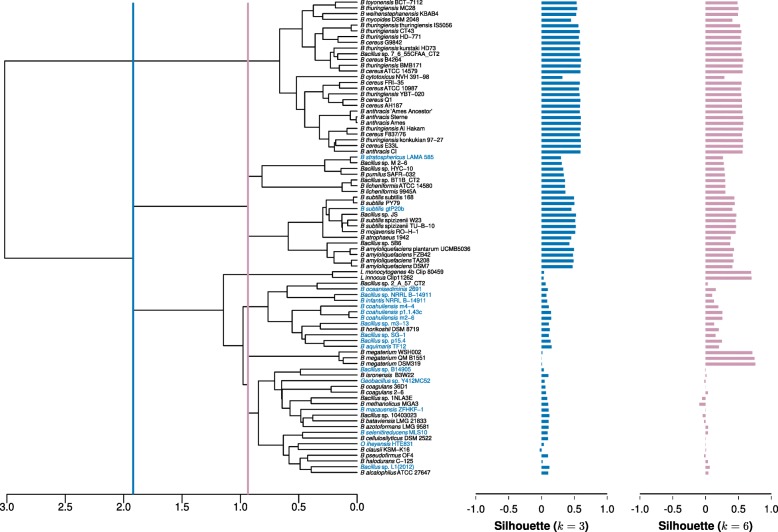
Cluster analysis using Jaccard’s distance (Jd) based on Figfams. The dendrogram was obtained using the Ward clustering method. Silhouette values are shown as blue and pink bars for *k*=3 and *k*=6, respectively. The blue and pink lines indicate the heights where the dendrogram was cut (*k*=3 and *k*=6, respectively). The names of the strains shown in light blue correspond to *Bacillus* isolated from aquatic environments

For both COG and Figfam based analyses, the best hierarchical dendrogram was obtained using the Ward method (COG agg. coeff. = 0.91; Figfam agg. coeff. = 0.92). Both COG and Figfam based hierarchies contained three main clusters, two of which were similar to the largest clades seen in the phylogenomic tree: the *B. cereus* and *B. subtilis* clusters. The third cluster contained the rest of mainly halophilic and alkaliphilic *Bacillus* (Figs. 4 and 5).

For COGs-based hierarchical dendograms, the *B. cereus* and *B. subtilis* clusters showed positive Silhouette values, while the third group exhibited from poor to a few negative values. To establish the number of clusters (*k*) in the hierarchies, the hierarchies were cut at different thresholds, and the resulting clusters evaluated by their Silhouette values. We started computing the Silhouette values for the three main groups by taking as limit the well supported clusters of *B. cereus* and *B. subtilis*. The analyses went up to 10 clusters (*k*=10) before disrupting the *B. subtilis* limiting cluster (Fig. 4). Interestingly, one of these 10 clusters consisted of seven aquatic *Bacillus* species that were located in different clades in the phylogenomic reconstructions (Figs. 2 and 3).

The hierarchical clustering using Figfam-based Jaccard distances also presented two groups with good Silhouette values, with a third group showing low but positive Silhouette values (Fig. 5). Cutting the dendrogram to maximize the Silhouette values, resulted in six clusters before disrupting the two main clusters. At this point 10 aquatic *Bacillus* species joined in a single cluster that included only two non-aquatic members. This result is remarkable since the aquatic *Bacillus* species appear to have polyphyletic origins (Figs. 2 and 3) and some of them were still separated in the COG-based Jaccard distance clusters (Fig. 4).

Since these clusters are based on shared gene content, the results suggest that the aquatic *Bacillus* species might share some functions beyond what would be expected from their polyphyletic origins. This functional convergence might be due to gain or loss of particular genes, or, in other cases, to the expansion of some gene families.

### Content-based genome clusters suggest environmentally-driven functional homoplasy

**Fig. 6:**
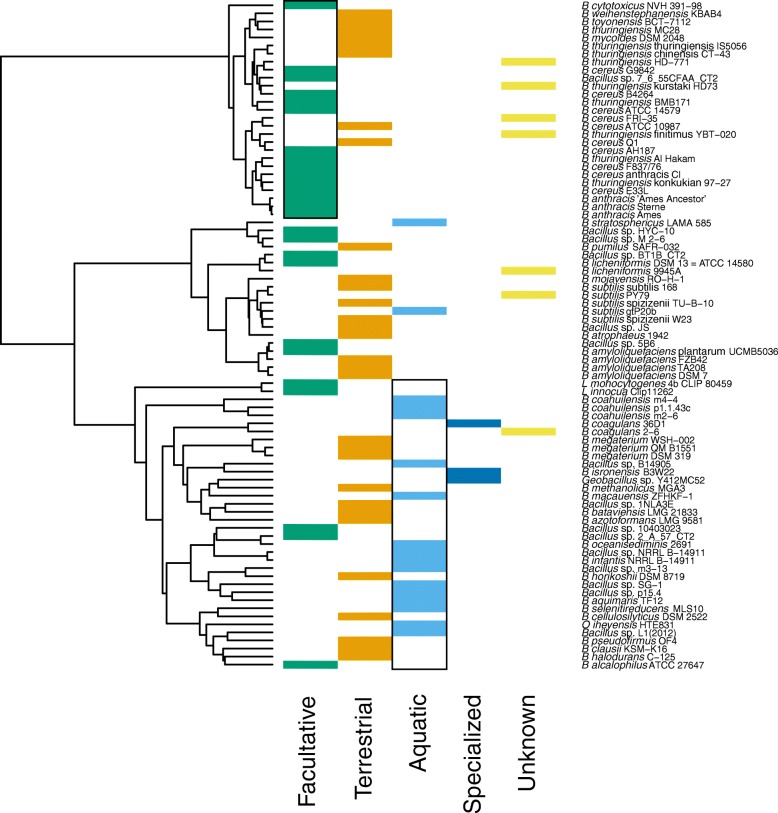
Significant association between the natural environment and the COG-based clustering groups. The *p*-values were calculated based on the hypergeometric distribution of the groups obtained from the hierarchical clustering with COGs. The significant associations are indicated by black squares. Facultative corrected *P*=1.7×10^−03^, Aquatic corrected *P*=5.6×10^−04^

**Fig. 7:**
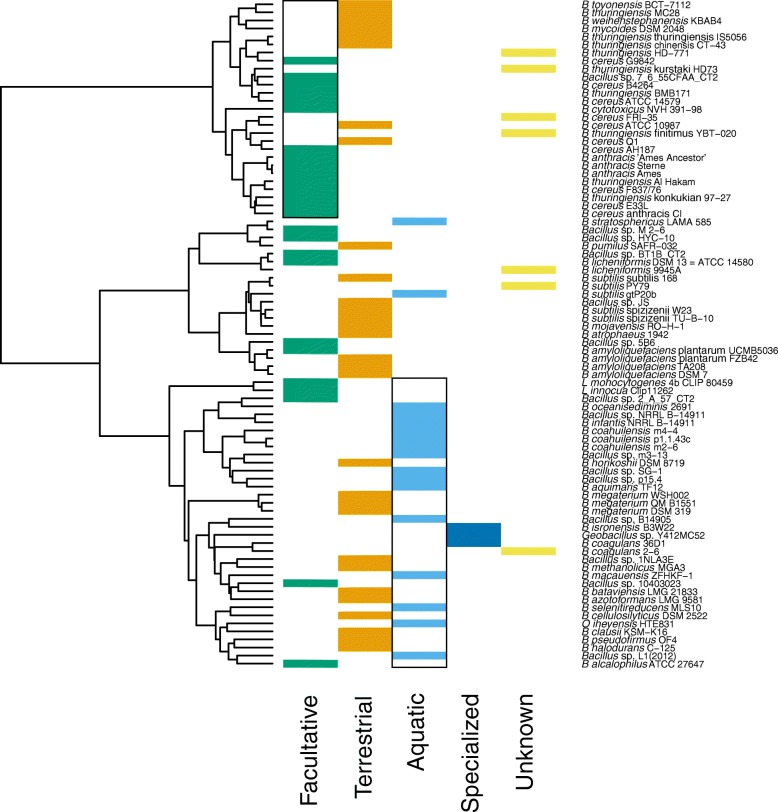
Significant association between the natural environment and the Figfam-based clustering groups. The *p*-values were calculated based on the hypergeometric distribution of the groups obtained from the hierarchical clustering with Figfams. The significant associations are indicated by black squares. Facultative corrected *P*=1.8×10^−03^, aquatic corrected *P*=9.0×10^−05^

To investigate associations between the environment and the COG and Figfam-based clusters, we calculated *p*-values based on the hypergeometric distribution using the clusters observed in the dendrograms obtained with COGs and Figfams and the environment of each organism (see “Methods” section). The similar results using both COGs and Figfams suggest that these are meaningful (Figs. 6 and 7). Interestingly, each main group in the dendrogram is associated with a main natural environment except for the *B. subtilis* group.

For both COGs and Figfams, we observed an association between the Facultative environment and the cluster containing the *B. cereussensu lato* group (Figs. 6 and 7). The species in this cluster grow saprophytically under rich nutrient conditions. It has been proposed that some members of this cluster can develop a symbiotic relationship with invertebrate hosts, occasionally developing a pathogenic lifestyle [55]. To date, there is little data available about the ecology of the terrestrial strains in this cluster.

Another association was found between the Aquatic genomes and the third cluster comprising seven of the nine phylogenetic clades observed in the phylogenomic reconstruction (Fig. 2). It is important to note that 15 out of 18 possible aquatic *Bacillus* were found inside this cluster (Figs. 4 and 5). Within this cluster we observed a subgroup consisting of nine and 11 aquatic *Bacillus*, depending on whether COGs or Figfams were used (Figs. 6 and 7).

Since organisms depend on their metabolic and regulatory capabilities to survive in specific environments, it is reasonable to expect a relationship between gene functional content and the environment. Some studies have already shown a relationship between an organism’s metabolism and its environment [56, 57], while others have suggested the existence of environment-specific genes involved in metabolic pathways that are hypothesized to be responsible for bacterial adaptation [58, 59]. A comparative genomic analysis of plant-associated *B. amyloliquefaciens* and *B. subtilis* strains, against non plant-associated strains, suggested that the differences in their genomes occurred during their adaptation to different habitats [60]. To examine if the aquatic *Bacillus* species contain specific functions distinct from the species in the other clusters, we compared the core functions among all clusters.

To investigate the presence of group-adaptive COGs, we determined the possible adaptive COGs. That is, those genes shared only between the organisms in the same cluster. We then compared their potential functions with those observed in other clusters. For this analysis we used the three main clusters from the Jaccard distance hierarchy and a matrix representing COG frequencies. We found that the *B. cereus* cluster contained the largest number of potentially adaptive COGs, 196, followed by the *B. subtilis* cluster with 106 and the aquatic *Bacillus* cluster with 29.

The aquatic *Bacillus* contained 29 putatively adaptive COGs, fewer than other clusters (Table 3). The overrepresented COGs belonging to category [G] and some in the category [E] consisted of sequences related to the transport of dissolved organic carbon (DOC). These genes were found in a metagenomic study of genes expressed by bacterioplankton [61]. The three COGs found in the [G] category (COG1653, COG1175, COG0395) were generic carbohydrate transporters, and the COGs in the [E] category (COG747 and COG0601) were oligopeptide transporters.

**Table 3:** Putative adaptive COGs found in the Aquatic *Bacillus* group

COG Category	COG Id	Description
EP	COG0601	ABC-type dipeptideoligopeptidenickel transport systems, permease components
E	COG0624	Acetylornithine deacetylaseSuccinyl-diaminopimelate desuccinylase and related deacylases
E	COG0747	ABC-type dipeptide transport system, periplasmic component
E	COG1703	Putative periplasmic protein kinase ArgK and related GTPases of G3E family
G	COG0395	ABC-type sugar transport system, permease component
G	COG1175	ABC-type sugar transport systems, permease components
G	COG1653	ABC-type sugar transport system, periplasmic component
HE	COG0111	Phosphoglycerate dehydrogenase and related dehydrogenases
I	COG0183	Acetyl-CoA acetyltransferase
I	COG1250	3-hydroxyacyl-CoA dehydrogenase
I	COG1884	Methylmalonyl-CoA mutase, N-terminal domainsubunit
I	COG1960	Acyl-CoA dehydrogenases
I	COG2185	Methylmalonyl-CoA mutase, C-terminal domainsubunit (cobalamin-binding)
J	COG4108	Peptide chain release factor RF-3
M	COG1215	Glycosyltransferases, probably involved in cell wall biogenesis
O	COG0695	Glutaredoxin and related proteins
O	COG1765	Predicted redox protein, regulator of disulfide bond formation
P	COG0607	Rhodanese-related sulfurtransferase
Q	COG0179	2-keto-4-pentenoate hydratase2-oxohepta-3-ene-1,7-dioic acid hydratase (catechol pathway)
R	COG0388	Predicted amidohydrolase
R	COG0673	Predicted dehydrogenases and related proteins
R	COG1647	Esteraselipase
S	COG2966	Uncharacterized conserved protein
S	COG3610	Uncharacterized conserved protein
T	COG0784	FOG: CheY-like receiver
T	COG2199	FOG: GGDEF domain
T	COG2200	FOG: EAL domain
T	COG2202	FOG: PASPAC domain
V	COG0841	Cationmultidrug efflux pump

The overrepresented COGs in the [I] category also suggested metabolic differences between clusters. For instance, the aquatic *Bacillus* species contained COG0183 (acetyl-CoA acetyltransferase), COG1250 (3-hydroxyacyl-CoA dehydrogenase) and COG1960 (Acyl-CoA dehydrogenase), which are involved in the degradation of lipids. The overrepresentation of some COGs involved in lipid degradation was previously found in the oligotrophic marine bacterium *Sphingopyxis alaskensis* RB2256 [62]. In contrast, the overrepresented COGs in the [I] category, within the *B. cereus* and *B. subtilis* clusters, are involved in lipid biosynthesis (Additional file 5: Table S3).

The fact that the functional gene content, represented in either COG or Figfam categories, were present among the members of a cluster and not shared with the members of other clusters would suggest specialization via the accessory genome. This specialization could be related to the environmental conditions that these organisms face. Importantly, the groups isolated mainly from terrestrial environments contain the most group-specific COG and Figfam categories. We speculate that organism living in a complex environment (i.e. soil, where the conditions can change drastically), would need a diverse battery of functions to help them survive, unlike aquatic organisms which might face a less heterogeneous environment.

It should be noted that the analysis of the core and accessory genome for the 83 complete and draft *Bacillus* genomes did not include genomes with plasmids. While draft genomes may contain some plasmid information, this information may be incomplete. The most extensively studied *Bacillus* species regarding plasmids is the *B. cereus sensu lato* group. Zheng et al. [63] analyzed twenty *B. cereus sensu lato* genomes, uncovering striking data of the gene contribution of plasmids in the *B. cereus sensu lato* pangenome. There were no “plasmid-core genes” in the combined plasmids sequences of the 20 strains. However, they found that the plasmids and chromosomes share the same functional gene pool. All of the COG categories represented in chromosomes could also be found on plasmids. Noteworthy, even category (V), defence mechanisms, is not higher in plasmids than in the chromosome. The authors suggest that HGT can move different types of genes that can aid in adaptation and that these genes can integrate into chromosomes or plasmids. Some genes may eventually be assimilated into the chromosome pool or get lost. With an increasing effort to sequence plasmids from environmental *Bacillus* spp. strains (for example [64]), we can expect that enough information will be obtained to evaluate plasmid contributions to environmental adaptation.

### *Bacillus* and their environments

The genomes selected for our study belong to strains that were collected from different environments around the world. Unlike pathogenic strains, for which there is more information on the events of the collection, most strains were assumed to have their habitat in the place where they were isolated. However, *Bacillus* species are capable of producing resistant spores. Thus, ecological studies with these organisms are challenging, since it is possible that their spores could simply survive anywhere, and that finding a *Bacillus* strain in a given environment might not be informative about their niche. However, our results and the results of others show a strong correlation between the place of isolation and metabolic capabilities. For example, previous work has found that the antagonism between *Bacillus* strains is isolation-site specific, at meters of distance between communities. This suggests that the interaction among bacteria, necessarily in a vegetative state, resulted in a selection for *Bacillus* species that could survive interactions within each community [65]. Another study showed that *Bacillus* strains recovered from soil and sediment possessed distinct capabilities for phosphorus utilization [66]. Finally, in a collection of *Bacillus* strains from different environments in India, a strong correlation was observed between tolerance to different pH, temperature, and salt concentrations versus the environmental features of the site of isolation [67].

## Conclusions

The evolutionary analysis of the *Bacillus* clade suggests that the aquatic *Bacillus* species have polyphyletic origins. Even though the aquatic *Bacillus* group proposed by Alcaraz et al. [4], did not hold in our analysis, we still observed a clade consisting of *Bacillus* isolated from distinct aquatic environments. The analyses based on functional content, represented as COG or Figfam categories, suggest that organisms in the *Bacillus* genus share functional content presumably selected by the environment from which they were isolated. Therefore, organisms isolated from similar environments might share genes beyond those expected from their phylogenetic origins, thus suggesting homoplasy at the gene-content level.

## Additional files

Additional file 1 **Supplementary Table 1.** Natural environment classification of the organisms used in this study. (TXT 6 kb)

Additional file 2 **Supplementary Table S2.** General information about the organisms used in this study. (TXT 5 kb)

Additional file 3 **Figure S1.** Maximum Likelihood phylogenetic reconstruction based on the 16S rRNA. The branch colors correspond to clades observed in the Core Genome Tree. The names of species corresponding to *Bacillus* isolated from aquatic environments are shown in blue. The bootstrap values are indicated as dots. Bootstrap values ≥80*%* are shown. (PDF 22 kb)

Additional file 4 **Figure S2.** Phylogenetic tree based on 11 phylogenetic marker sequences. The branch colors correspond to clades observed in the Core Genome Tree. The *Bacillus* species shown in blue were isolated from aquatic environments. The bootstrap values are indicated as dots. Bootstrap values ≥80*%* are shown. (PDF 23 kb)

Additional file 5 **Table S3.** List of putative adaptive COGs in the *B. subtilis* and *B. cereus* groups. (TXT 16 kb)

## Abbreviations

COG: Clusters of Orthologous Groups
*GSS*: Genomic similarity score
ML: Maximum likelihood
MLST: Multi locus sequence typing
RBH: Reciprocal best hits
