# Supplementary figures and images for: Environmentally-driven gene content convergence and the Bacillus phylogeny
Source: BMC Evol Biol. 2018 Oct 3;18:148. doi: 10.1186/s12862-018-1261-7 (PMC6171248; doi:10.1186/s12862-018-1261-7)

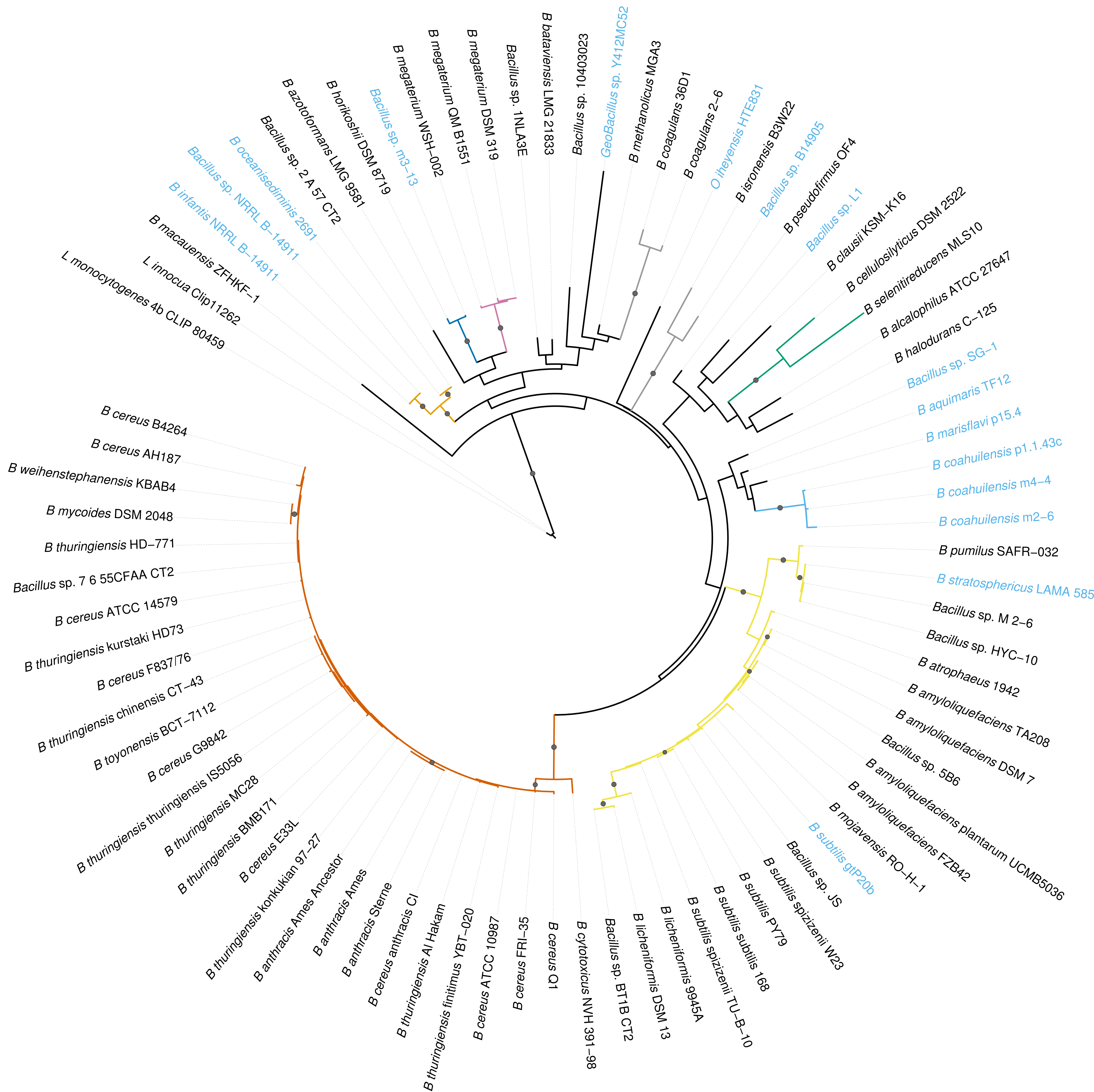

Supplement: Supplementary file 3 — Figure S1. Maximum Likelihood phylogenetic reconstruction based on the 16S rRNA. The branch colors correspond to clades observed in the Core Genome Tree. The names of species corresponding to Bacillus isolated from aquatic environments are shown in blue. The bootstrap values are indicated as dots. Bootstrap values ≥80% are shown. (PDF 22 kb) [file 12862_2018_1261_MOESM3_ESM.pdf]

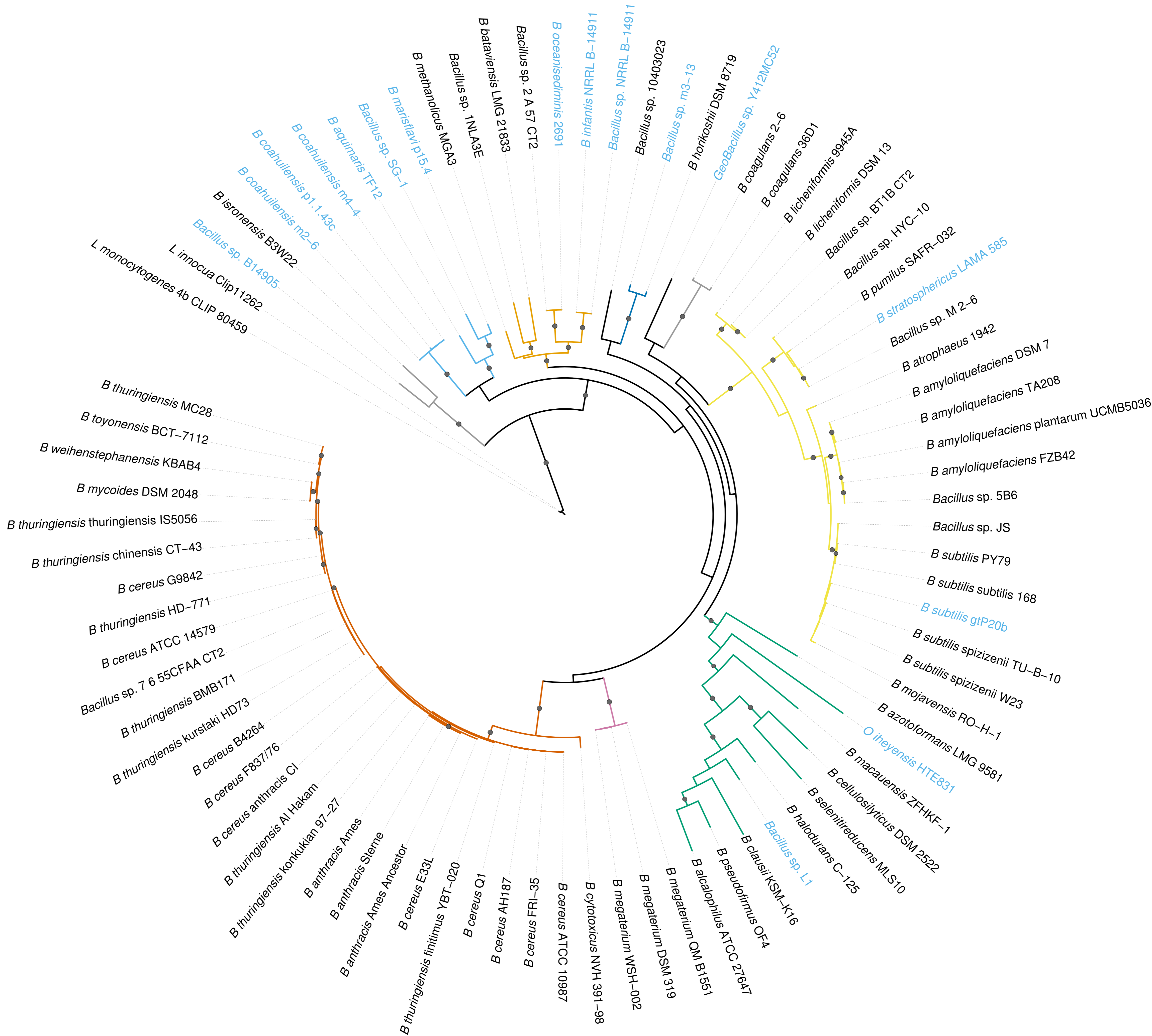

Supplement: Supplementary file 4 — Figure S2. Phylogenetic tree based on 11 phylogenetic marker sequences. The branch colors correspond to clades observed in the Core Genome Tree. The Bacillus species shown in blue were isolated from aquatic environments. The bootstrap values are indicated as dots. Bootstrap values ≥80% are shown. (PDF 23 kb) [file 12862_2018_1261_MOESM4_ESM.pdf]
